# Life in a slum neighborhood of Addis Ababa, Ethiopia: morphological facts and their dysfunctions

**DOI:** 10.1016/j.heliyon.2021.e07139

**Published:** 2021-05-29

**Authors:** Edomgenet Hiba Issa

**Affiliations:** Department of Sociology, Mizan-Tepi University, SNNPR, Ethiopia

**Keywords:** Demography, Positionality, Being a Slum as a Relational Construct, Psychological distress, Economic and social cost, Gender Dimension of Dysfunctions

## Abstract

**Objectives:**

(1) To explore morphological facts that typically characterize life in *Setegn Meda* slum neighborhood. (2) To identify dysfunctions of those morphological facts. (3) To discuss some of the differences and similarities between the study area and other forms of a slum in Addis Ababa as outlined by Alemayehu (2008) based on selected morphological facts.

**Methods:**

The study adopted a qualitative case study research design rooted in a pragmatic constructivist approach to a case study. Information was gathered through semi-structured interviews and field observation, and then analyzed using a deductive thematic analysis technique.

**Results and conclusion:**

This study found that the morphological facts that typically characterize life in *Setegn Meda* slum neighborhood are: Dilapidated Housing; Limited Access to Infrastructure; Congested Settlement; Positionality; and Being a Slum as a relational Construct. This study also found that these morphological facts have their inherent dysfunctions: Physical Health Risk; Psychological Distress; Economic Cost; and Social Cost. Results further show that the dysfunctions of some morphological facts have a gender dimension where women suffer more. The difference and similarities between the study area and other forms of a slum in Addis Ababa as outlined by Alemayehu (2008) were discussed based on selected morphological facts. The discussion suggests that let alone the homogeneity of slums at the global level, slums are rather heterogamous even at a national/local level. The discussion has also highlighted that the classification of slums in Addis Ababa as proposed by Alemayehu (2008) for one thing is not exhaustive and needs some modifications.

**Implications:**

The study is expected to hold significance both at empirical and theoretical levels. Some of the empirical significances of this study are: it challenges the conventional categorization of slums in Addis Ababa; it provides a thick description of morphological facts in a slum neighborhood and their dysfunctions to the inhabitants, and it brings the gender dimension of dysfunctions of morphological facts to the audience. Whereas, the theoretical significance could be drawn from how the study tried to make functionalism theoretically useful to the study of a slum habitat.

## Teaser

Demography; Positionality; Being a Slum as a Relational Construct; Psychological Distress; Economic and Social Cost; and Gender Dimension of Dysfunctions.

## 1 Introduction

Slums are a common attribute of urbanization and urban settlement patterns in the developing world (Gottdiener et al., 2018). The actual number of people living in slums grew to over 1 billion, with 80 percent attributed to three regions: Eastern and South-Eastern Asia (370 million), sub-Saharan Africa (238 million), and Central and Southern Asia (227 million). The total population of slum dwellers across the world is estimated to reach 3 billion by 2030 (Daniel, 2015).

The term ‘slum’ in conventional usages indicates ‘bad’ shelter (Gilbert, 2007). Commonly, the term may refer to settlements constituted by substandard houses and poor inhabitants (Gilbert, 2007). In practice, despite the efforts made to establish an objective definition, every city in the world seem to define slums in its own way (Gilbert, 2007).

In an attempt to outline an objective indicator of the slum, Cities Alliance defined slum as a settlement that lacks basics infrastructures (such as road, water, sanitation, etc.), open public spaces that can be used for social gatherings, and public safety (i.e. safe area for children to play) (Gilbert, 2007). But, later the definition was refined as a settlement where a substantial proportion of households are deprived of the five key indicators: access to improved water; access to improved sanitation; sufficient living area, the durability of housing; and access to secure tenure (UN-Habitat, 2003). These days, the UN and other international development organizations adhere to this definition. For the sake of its objectivity, this study also shares UN-Habitat's definition of the slum. Besides, for its objectivity, this study has also shared UN-Habitat's definition of the slum for its direct relatability to the subject matter of the Study-Morphological Fact.

Despite the acceptance and use of the term ‘slum’ by the United Nations, World Bank, and other international development organizations, academicians argue that the term is a dangerous word to use. Gilbert (2007), for instance, argues that the use of the word ‘slum’ has ramifications. Some of these could be: it is easily manipulatable by political entrepreneurs, land developers, and planners; it could implicate that slum clearance is the best solution to slums. Some observers have also pointed out that the term ‘slum’ harbors a feeling of dislike to exists along with a hope for a complete change (Marris, 1979; Yelling, 2012).

In the literature on slums, as in the case with the use of the term ‘slum’, the debate on heterogeneity/homogeneity of slum habitat is also quite observable. Many observers, including the UN and other development organizations, tend to portray all slums as bad and everyone living in them suffering from their grim realities. Politicians, planners, and the general public all tend to adopt the same view (Gilbert, 2007). According to the UN, slums in urban Africans, Asians, and Latin Americans are no longer exceptional (UN-Habitat, 2003). Accentuating the same point, Davis (2006), claims that much of the cities in today's world are slums.

Contrary to the conventional notion that depicts slums as homogeneous ‘badlands’, plenty of observers have also put forward their counter-arguments. For instance, from a study of seven different countries, Turner (1967), shows how slums are heterogeneous after discovering the peripheral slum dwellers have a higher socioeconomic status than the inner-city slum dwellers. Gilbert (2007), also argues that life in all slums is not homogenous and as dismal as that described by the UN and other studies. To capture the heterogeneity of slums, years ago, Charles Stokes has also tried to differentiate between ‘slums of hope’ and ‘slums of despair’ (Stokes, 1962).

Previous authors on slums in Ethiopia also appear to contribute to the debate on the homogeneity/heterogeneity of slums. On the one side, too many observers seem to claim that slums are the harshest place to live for mankind and call for prompt actions. The main points echoed by such observers were the fact that slums are synonymous with deteriorated houses, unsanitary outlooks, and scarcity of basic infrastructure (Biniyam et al., 2018; Getachew et al., 2007; Sanbata et al., 2014; Tolossa, 2010; and UN-Habitat, 2007a, b). On the other side, some authors have tried to capture some of the positive aspects of life in slum neighborhoods. These studies maintain that one of the good things slum dwellers have was strong informal social networks (Abebe and Hesselberg, 2013; Abegaz, 2014; Samson, 2014).

According to Alemayehu (2008), slums of Addis Ababa can be categorized into three types: (1) Non-planned old inner-city settlements, dominated by *kebele* housing and occupied by tenants with some tenure rights. (2) Informal peripheral squatter settlements, built on vacant land with little or no infrastructure and with uncertain or no tenure rights. (3) Inner-city squatters with no tenure rights. These are plastic houses that are built occupying parts of public parks, squares, vacant open spaces, and as attachments to streets side fences. A closer look into previous studies on slums in Ethiopia could unveil the fact that the vast majority of the studies were conducted on the so-called old inner-city slum neighborhoods of Addis Ababa Metropolitan city. It can particularly be noticed that, as though there were no other slum neighborhoods in the city, studies after studies were conducted on inner-city slums such as *Arat kilo*, *Piazza*, *Merkato*, and *Ledeta* areas, etc (Alemayehu, 2008; Kloosterboer, 2019; Megento, 2013; Teferi and Newman, 2017; UN-Habitat, 2007b; Weldeghebrael, 2020; Yntiso, 2008). Whereas some have paid their share of interest on informal peripheral settlements (Abagissa, 2019; Larsen et al., 2019; Lirebo, 2006; Wubneh, 2013).

However, from a simple tour throughout Addis Ababa, one could learn that there exist settlements that qualify UN-Habitat's standard for slums yet neither are non-planned old inner-city settlements nor informal peripheral squatter settlements nor inner-city squatter settlement. It could be, thus, noticed that such a general tendency of focusing the researcher's lens mainly either on the inner-city slum neighborhoods or on informal peripheral settlement seems to have left other slum neighborhoods less explored if not unexplored at all.

The scarcity of knowledge on slums that are neither non-planned old inner-city nor informal peripheral squatter nor inner-city squatter settlement seems to harbor both risk and opportunity. The risk is that city administrations may plan and try to execute a program that does/may not discriminate the diversity in slum neighborhoods. Here, it is worth mentioning that many countries have been observed failing costing billions of dollars and throwing beneficiaries (in this case slum dwellers) to greater life challenges whenever trying to put in action a homogenizing plan to a heterogeneous population. With such limitations of knowledge, the same fate may befall cities in Ethiopia and their supposed beneficiaries. The gap could also be seen as an opportunity. An opportunity for researchers and academia to bring forward questions whose answers could frame and enlighten debates on slums. This study, thus, could be perceived as a researcher's curiosity conceived by the risk that lurks around the gap in the literature on slums that are neither non-planned old inner-city nor informal peripheral nor inner-city squatter settlement. Then delivered in and through the opportunity that unfolds beneath previous authors' linear focus on either the inner-city slums or informal peripheral settlements.

Informed by the debate on homogeneity/heterogeneity of slums, this study attempts to explore Morphological facts of a slum habitat that is neither non-planned old inner-city nor informal peripheral nor inner-city squatter settlement and their dysfunctions to the lives of the inhabitants. Three specific objectives were framed to guide this study. (a) To explore morphological facts that typically characterize life in *Setegn Meda* slum neighborhood. (b) To identify dysfunctions of those morphological facts. (c) To discuss some of the differences and similarities between the study area and other forms of a slum in Addis Ababa as outlined by Alemayehu (2008) based on selected morphological facts.

## 2 Theoretical framework

Functionalism is a theoretical perspective with a vast niche and enormous influence in sociology in general and in sociological theories in particular. The root of functionalism can be traced back to the seminal works of the founding fathers of sociology such as Herbert Spence and Emile Durkheim (Ritzer, 2007). Early functionalist theories accentuate the contribution made by social arrangements to maintain and reproduce society and culture. But after WW II, in an attempt to fill the gaps, early functionalist theories were adopted and modified by Talcott Parsons and Robert Merton (Wason, 2007). The 1960 & the 70s were periods when functionalists had lost their hegemony to conflict and symbolic interactionist perspectives. Then no later than a decade, in the 1980s, functionalism was revived by a new wave of neo-functionalists such as Jeffrey Alexander, Nikolas Lehman, and Jürgen Huberman (Wason, 2007). Today, under the umbrella of functionalism, diverse strands of functionalist theorizing and abstraction could be found (Isajiw, 2013; Judge, 2012; and Mulkay, 2016). This study, thus, rooted its theoretical foundation only into two basic concepts drawn from the functionalist literature- *‘Morphological Fact’* and *‘Dysfunction’*.

Morphological fact is a concept drawn from Durkheim's theory of social facts. According to Durkheim, a social fact is a category of facts consisting of ways of acting, thinking, and feeling, external to the individual, and endowed with a power of coercion by means of which they control him (Durkheim, 1966). For Durkheim, a social fact could be classified in different ways. One way of classification is as a social fact of a physiological order (operative social fact) and social fact of morphological order (in this research sense, morphological fact). The social fact of a physiological order includes a society's legal code, religious beliefs, the concept of beauty, monetary system, ways of dressing, or its language. Whereas social fact of morphological order is often concerned with the demographic and material conditions of life and includes the number, nature, and relation of the composing parts of society, their geographical distribution, the extent and nature of their channels of communication, the shape and style of their buildings, and so forth. Durkheim argues that social facts are ‘sui generis’ realities above and beyond the individual and should not be reduced to economic, psychological, or physiological facts (Durkheim, 1966).

Though the term ‘morphological fact’ is not commonly used in sociological vocabularies, the general notion underlying the term seems to prevail in sociology in general and urban sociology in particular in the name of ‘space’. Space may constitute has different aspects. Some of these aspects are the space that surrounds the individual as an imaginary esoteric body, the private space such as a home that we use as a backstage in our everyday drama of life, the situated space that we use as a frontstage to perform the everyday life drama called social interaction, and the public spaces of where bigger social events carried out. Sociological research of space might investigate one or more of these dimensions (Wason, 2007).

It is Hall's seminal work, The Hidden Dimension (1966), that brought space as a sociological subject matter of study. For Hall, and other well-known theorists of space such as Lefebvre, space is a social construction that structures as well as structured by human agency (Wason, 2007).

Though their focus areas vary, many studies of space focus on urban settings. Few key examples are a study on contested public space and marginalization (Madanipour, 2004); a study on the homogenization of cultural aspect of public urban spaces and its impact on intercultural communication (Rahder and Milgrom, 2004); a study on disability and social responses in spatialized political economies (Kitchin, 1998); a study on the nexus between a spatial organization and social interactions (Gans, 1982); and a study on the nexus between human behavior and environment (Jones, 1984).

This study borrowed the concept of morphological fact for three reasons. The first reason was that, while holding its root in the functionalist theorizing, the concept ‘morphological fact’ appeared to capture most of the sociological dimensions of ‘space’. The second reason was to recognize and frame the physical conditions of life in a slum neighborhood as social facts of a morphological order (morphological facts). Whereas, the third reason was to understand and elaborate on the physical conditions of life in a slum neighborhood as *sui generis* realities that are irreducible to ecological, economic, and psychological factors.

The second concept drawn from the functionalist perspective is the notion of ‘Dysfunction’. The idea and study of the dysfunction of social facts are first introduced into sociology by Robert Merton. Unlike his predecessors, Merton theorized that social facts do not only have functions (positive contribution to the society) but also sometimes espouse dysfunctions (negative consequences) to certain individuals and population segments (Fine et al., 2003).

Borrowing the term dysfunction in Merton's sense and integrating it with Durkheim's conception of morphological fact had two purposes. One was to enable the researcher to frame the negative consequences (dysfunctions) of physical aspects of life in a slum neighborhood (morphological facts) within a sociological paradigm. The other was to conceptualize dysfunctions of morphological facts in a slum neighborhood as emergent properties of other social facts. In a sense, explainable through other social facts rather than through psychological, economic, or physical characteristics of the individuals residing in a slum neighborhood.

## 3 Methods

### 3.1 Design and philosophical orientation

The study adopted a qualitative case study research design. In terms of philosophical orientation, the study adhered to a constructivist's philosophical orientation towards the qualitative case study. More specifically, it adhered to Marriam's pragmatic constructivist approach to a case study (Harrison et al., 2017). Marriem's approach to qualitative case studies has significantly shaped this study. For instance, at the ontological level, the researcher shared Marriem's notion that reality is socially constructed. The goal of the research, therefore, was not to find out an objective universal law/truth as positivists wish to capture but rather to understand a context-bound socially constructed reality. The researcher also shared Marriem's pragmatic epistemological inclination. This could be seen in different instances. For instance, the case was determined based on research purpose. The specific objectives were drawn based on existing bodies of literature. The study was directed towards providing a rich and holistic description of the family and economy institutions in a slum neighborhood. The researcher was also committed to utilizing objective processes to collect, organize, and analyze bulky and abstract data.

### 3.2 Setting

**Figure 1:**
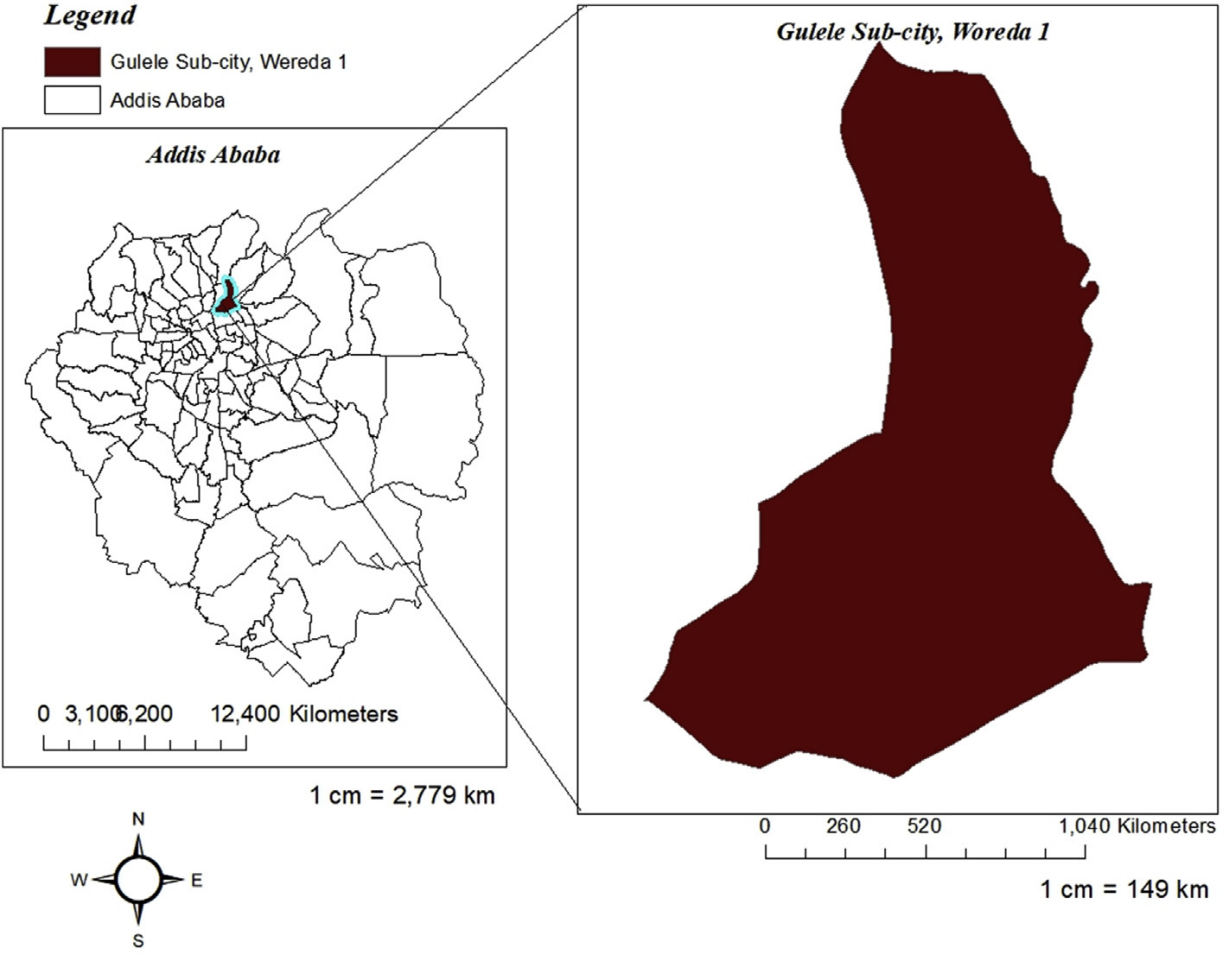
Locational map of gulele sub-city, woreda 1, along with Addis Ababa. This map was created using ArcGIS® software by Esri. ArcGIS® and ArcMap™ are the intellectual property of Esri and are used herein under license. Copyright © Esri. All rights reserved. For more information about Esri® software, please visit www.esri.com.

**Figure 2:**
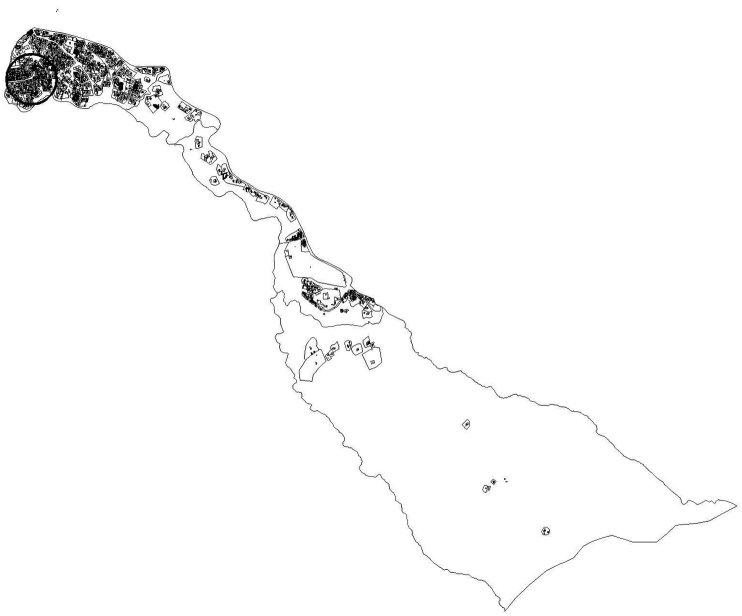
Spatial map of Woreda 1 (the study area is the one marked in circle). This map is accessed from Gulele Sub-City, Woredal 1, Administration Office, 2020. The map is the intellectual property of the sub-city and was used herein with permission.

This study was conducted on a slum neighborhood informally known as *Setegn Meda*. The slum neighborhood is located in *Woreda*^1^ *1* locally known as *Shero Meda*, Gulele Sub-city, Addis Ababa city, Ethiopia. The Woreda 1 administrative office estimates that around two hundred fifty (250) households live in this slum neighborhood. According to an expert from the sub-city land administration office, most of the residents live in *Kebele houses*^2^ while some have secured their piece of land following the necessary legal procedures. This expert has also mentioned that the neighborhood is an emerging slum settlement no more than 30 years old. Figures 1 and 2 show the locational context of the study site.

### 3.3 Data collection

Data were collected through semi-structured interviews and field observation. Two sets of people were interviewed. The first set of interviewees were residents. The question items prepared to guide the interview with residents were the following. *Tell me about your housing condition? What are the problems associated with your housing condition? Tell me about the main manifestations or attributes of basic infrastructures in your neighborhood? What are the problems associated with basic infrastructures in your neighborhood? Is there anything else you think we should know, or are there any questions we should have asked but didn't?*

The second set of interviewees were local government officials. The interview schedule prepared for local government officials consists of the following questions. *Tell me about housing conditions in Setegn Meda neighborhood? What are the problems associated with housing conditions in Setegn Meda neighborhood? Tell me about the main manifestations or attributes of basic infrastructures in Setegn Meda neighborhood? What are the problems associated with basic infrastructures in Setegn Meda neighborhood? Is there anything else you think we should know, or are there any questions we should have asked but didn't?*

This study has also made use of field observation. In the field observation, the role adopted by a researcher is important in that it constraints what can be observed (McKechnie, 2008). Raymond L. Gold has developed a typology of the role of a researcher in a field observation (McKechnie, 2008). By Raymond L. Gold's typology, the role of the researcher in this study could be described as ‘observer as a participant’. The actual data collection through field observation was done guided by an observation checklist.

### 3.4 Sampling

This study employed a purposeful sampling strategy. Sample selection criteria avoid a tunnel vision bias towards a single group (Maxwell, 2012). Thus, sampling criteria were set both for the selection of residents and the government officials. Sampling criteria for the selection of residents were: living in the study area for at least 5 years; owning either *kebele* or private housing unit; and having at least 1 child. Whereas, the criteria for the selection of the government officials were: working in the area for at least a year and working on a position that is directly related to the subject matter of this study.

### 3.5 Analysis

Interview and observation data were manually analyzed using a six-step deductive thematic analysis technique (Braun and Clarke, 2006). The analysis involved the following activities. First, getting familiar with the data. This involved transcription and translation of data into the English language and reading and re-reading the data. Two, generating initial codes. Initial codes were generated by coding interesting features of the data in a systematic fashion across the entire data set having the specific objectives and theoretical framework in mind. Then, data extracts relevant to each code were collated. Three, searching for themes. Themes were searched in and through the entire data set. This step mainly involved collating codes into potential themes and gathering all data relevant to each potential theme. Table was used as a tool to organize and present themes, sub-themes, and codes. In this step, the analyst has tried to think about the relationship between codes, themes, and levels of themes. Four, reviewing and refining themes and sub-themes. Five, defining, naming, and analyzing themes. In this step, the analyst clearly defined and named themes and sub-themes, conducted detailed analysis for each theme, and wrote a detailed analysis for each theme by identifying the story that each theme tells. Six, producing a report. The report consisted of two parts: analysis and discussion. The analysis part was made up of the analyst's narrative and illustrative data extracts. The discussion part constituted the analyst's narrative and findings of previous researches.

### 3.6 Ethical consideration

Ethical approval was received from Mizan-Tepi University, the Department of Sociology, ethical review committee. Before conducting the study, potential participants were informed about the objectives and procedures of the study and the assurance for anonymity of their identity and confidentiality of the data. Then, only those who volunteered to participate and signed an informed consent form were interviewed.

## 4 Results

### 4.1 Demographic information of participants

A total number of twenty-one (21) household heads and four (4) local government officials had participated in this study. The government officials were experts each recruited from Gulele Sub-City, Woreda 1 Administration, Municipal, Housing and Urban Development, and Social Affairs Office respectively. As for the household heads, Table 1 presents demographic information of the participants.

**Table 1:** Demographic information of household heads who participated in this study.

No.	Pseudonym	Gender	Age	Academic Background	Family Size	Ethnicity	Religion
1	Lema	Male	52	Only reading & writing	4	Oromo	Orthodox
2	Abiy	Male	42	Only reading & writing	1	Amhara	Orthodox
3	Senayit	Female	35	Only reading & writing	2	Amhara	Orthodox
4	Samrawit	Female	39	Only reading & writing	3	Amhara	Orthodox
5	Hana	Female	45	Only reading & writing	3	Other	Orthodox
6	Megersa	Male	42	Only reading & writing	2	Other	Protestant
7	Mohamed	Male	41	Only reading & writing	5	Oromo	Muslim
8	Tsehay	Female	47	Only reading & writing	5	Oromo	Orthodox
9	Hagos	Male	41	Only reading & writing	5	Tigre	Protestant
10	Medina	Female	39	Only reading & writing	3	Amhara	Orthodox
11	Meles	Male	52	Only reading & writing	2	Tigre	Orthodox
12	Terhas	Female	41	Only reading & writing	3	Tigre	Orthodox
13	Musa	Female	47	Diploma	4	Guraghe	Orthodox
14	Beza	Female	28	High school complete	1	Guraghe	Orthodox
15	Meaza	Female	42	High school complete	4	Guraghe	Orthodox
16	Nigist	Female	45	High school complete	5	Amhara	Muslim
17	Ketema	Male	36	High school complete	3	Oromo	Muslim
18	Dergu	Male	38	High school complete	3	Amhara	Orthodox
19	Bulcha	Male	39	High school complete	3	Amhara	Protestant
20	Abezash	Female	40	Diploma	2	Other	Protestant
21	Birtukan	Female	67	Diploma	2	Amhara	Protestant

### 4.2 Morphological facts

Five major themes emerged from the analysis of interview and observation data about morphological facts. These were: Dilapidated Housing; Limited Access to Infrastructure; Demography; Positionality; and Being a Slum as a relational Construct. Table 2 presents the themes, sub-themes, and codes that emerged concerning morphological facts.

**Table 2:** Themes, sub-themes, and codes related to morphological facts.

Themes	Sub-themes	Codes
Dilapidated Housing	Sub-standard Construction Material	Mud wall and floor Corrugated iron roof Weather-beaten wall Roofs and walls with a punctured plastic sheet Scruffy and smudgy fabric ceiling
	Non-proportional Family Size and Number of Bedrooms Ratio	Perceived inadequacy of bedrooms as compared with family size
	Unhygienic Setting	Dirty toilet The proximity of toilet to bedroom and dining area No separate kitchen and bedroom Lack of appropriate liquid waste disposal mechanism
Limited Access to Infrastructure	Undependable Access to Basic Infrastructures	Undependable access to electric power Undependable access to tap water
	Inadequate Access to Basic Infrastructures	Inadequate access to municipal solid waste facilities and human resources. Solid wastes temporarily stored in an open space
Demography	Density	Houses built so close to each other Absence of open public spaces Narrow roads and alleys
	Homogeneity	Christian majority Amhara ethnic majority
Positionality	Relative Distance from Nucleuses of the City	Traveling relatively long distance to access essential social services (market, school, church, government organizations)
	Relative Distance from Center of the City (CBD)	Traveling relatively long distance to access essential social service (mega-market)
	Geographic Location in the City	Located in the northern part of the city Bounded by a mountain range
Being a Slum as a Relational Construct	Being a Slum to the City Administration	A place that comes last when it comes to the provision of public services Displacement in the name of slum clearance
	Being a Slum to Non-Slum Dwellers	A place where economically, socially, and morality low standard people live
	Being a Slum to the Inhabitants	Unfavorable to settle and raise children A transitionary settlement to life in a better place

#### 4.2.1 Dilapidated housing

A thematic analysis of interview and observation data showed that dilapidated housing condition is one of the morphological facts that typically characterize life in the study area. The thematic analysis also showed that a conceptual definition of dilapidated housing conditions embedded in and through the data sets has three dimensions.

**Figure 3:**
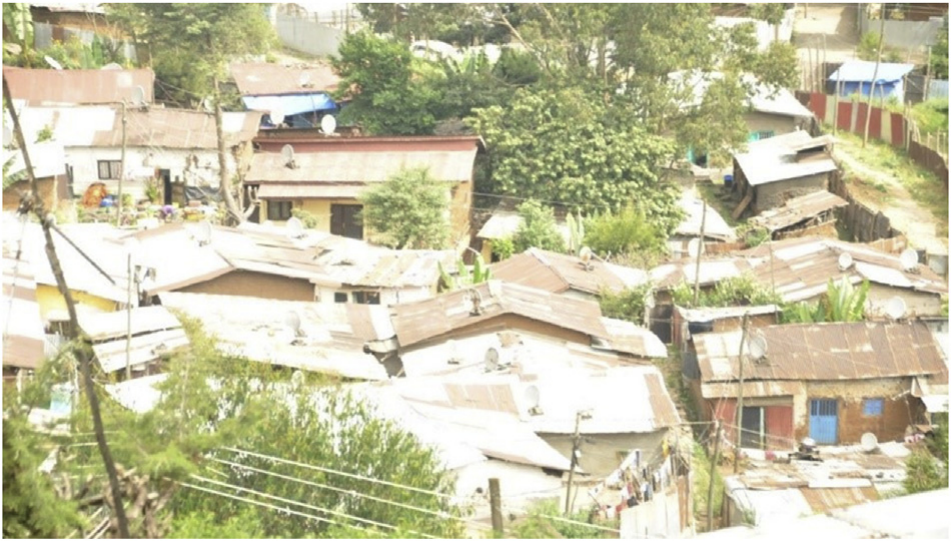
A panoramic view of exterior features of most houses in the study area. This image was taken during field observation in February 2020.

The first dimension of dilapidated housing was *sub-standard construction material*. The data from field observation indicated that many houses around the study area were made up of mud walls and corrugated iron roofs. The roofs of most of these houses were rusted whereas the mud walls were weather-beaten. During the field observation, some roofs and walls were seen with punctured plastic sheets. Relatedly, one government official (an expert from Gulele Sub-City, *Woreda* 1, Housing and Urban Development Office), told the researcher that the local government is well aware of the fact that most of the houses in the study area are constructed from sub-standard materials. Figure 3 shows the exterior of most houses in the study area.

The field observation has also made clear that the most common characteristic of the interior of houses in the study area were: roofs nearer to the ground with old, scruffy, and smudgy fabric ceilings that a standing average-height man could touch with stretched arms; walls decorated with plastered newspapers, and mud-floor covered with plastic sheets.

The second dimension of dilapidated housing conditions was *non-proportional family size and number of bedroom ratio*. The interview held with residents indicated that the family size of most households was non-proportional to the available number of bedrooms. Family size per bedroom data of some of the informants (residents) were: interviewee #1 (5/2), interviewee #2 (4/1), interviewee #3 (4/1), interviewee #4 (3/1), and interviewee #5 (5/1). Informants were also seen sharing perceived inadequacy of bedrooms as compared with family size. For instance, an informant said, “*I have three kids and one bedroom. It is hard to live in such a way.” (Hana*).

The third dimension of dilapidated housing conditions was an *unhygienic setting*. The field observation and interview data indicated that the general condition of houses in the study area was unhygienic. The houses were found out to be unhygienic for four reasons. First, toilets in most of the houses were shabby small rooms where holes dug in the ground and covered with wooden bars where one can hide from others but cannot hide from seeing what is in the hole. Toilets are also seen lacking water pipes or any other ways of accessing them. Flies, pungent smells, and very close proximity to bedrooms and dining areas were other attributes that characterize toilets in the studied slum neighborhood. Second, insects (fleas) and rodents (rats and mice) were found out to be residents of houses in the observed slum neighborhood as the people were. During the interview, every household reported that rats have always been in their houses. Relatedly, during field observation, the data collector had heard rats roaming in the ceilings of three of the observed houses. Third, most of the houses had hardly separated the kitchen and bedrooms. Besides, kitchens in most of the houses were found not that much far from the fly-infested toilets mentioned before. Fourth, most of the observed houses happened to be lacking appropriate liquid waste disposal mechanisms. The liquid wastes from those houses were seen disposed of in open spaces where children play and women carry out their daily chores.

#### 4.2.2 Limited access to basic infrastructures

Thematic analysis of interview and observation data revealed that the second morphological fact that characterizes life in the study area is limited access to basic infrastructures. Under this theme, two sub-themes had emerged: *Undependable Access to Basic Infrastructures* and *Inadequate Access to Basic Infrastructures*.

The field observation showed that most households in the neighborhood had installed electric power cables and some had installed private tap water pipelines. Interviewees (residents) however complained in unison about how their access to electric power and water had been undependable. According to the informants (residents), power blackout for hours on a daily basis was common. Informants (residents) also reported that the power blackout may sometimes last for a week. Though informants (residents) had not deemed access to private tap water as severely unreliable as the access to electric power was, they mentioned that they had been experiencing fluctuation on a regular basis. Case in point, an informant (resident) told, *“We might last for three weeks with water pipelines devoid of a water droplet”. (Medina).* Another informant (resident) also said, *“After a power blackout, you just know the power has come back again when you hear a cheering out loud voice throughout the neighborhood. The shouting tells how much we missed it”. (Melese*).

**Figure 4:**
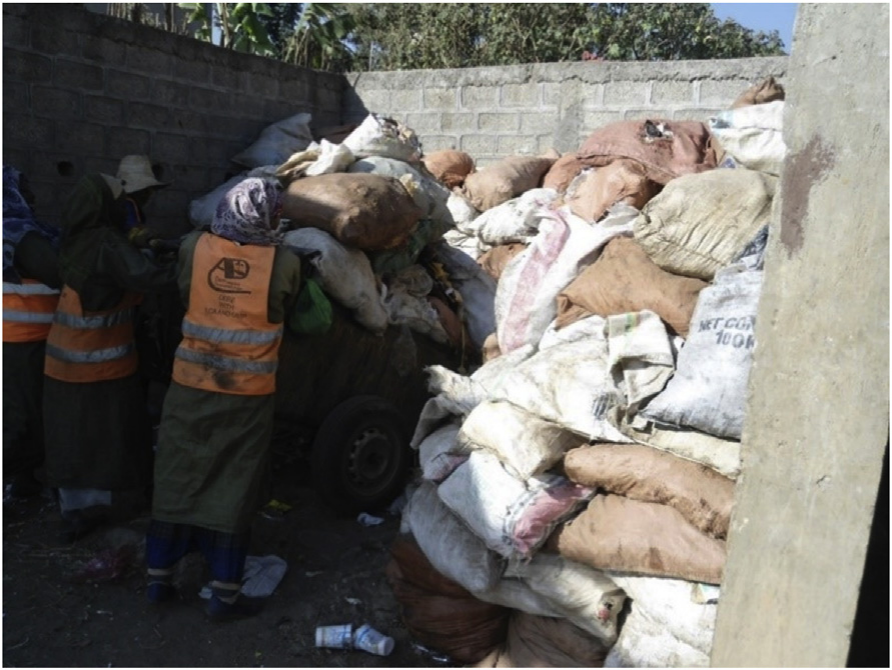
An open space that serves as a temporary storage area for solid wastes. This image was taken during field observation in February 2020.

The field observation and interview also showed that, among basic infrastructures in the study area, municipal solid waste facilities and human resources were inadequately available. An informant (resident) Mentioned, *“We have only two medium-size waste containers... only two women are often observed doing the cleaning job.” (Nigist)*. Solid wastes were also observed temporarily stored in open spaces. Figure 4 displays an open space that serves as a temporary storage area for solid wastes.

#### 4.2.3 Demography

**Figure 5:**
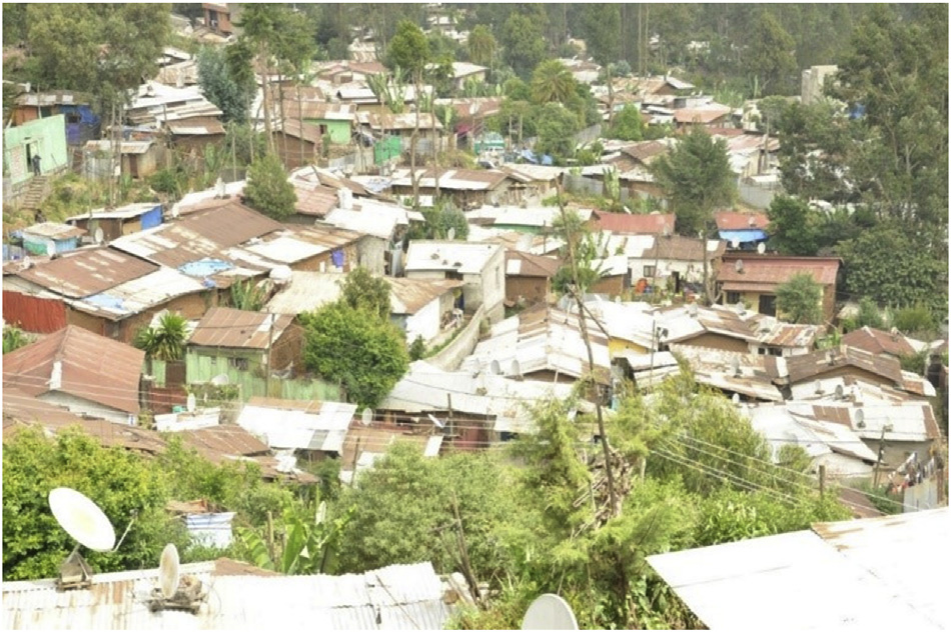
A panoramic view of the study area. The image shows how congested the study area is. This image was taken during field observation in February 2020.

**Figure 6:**
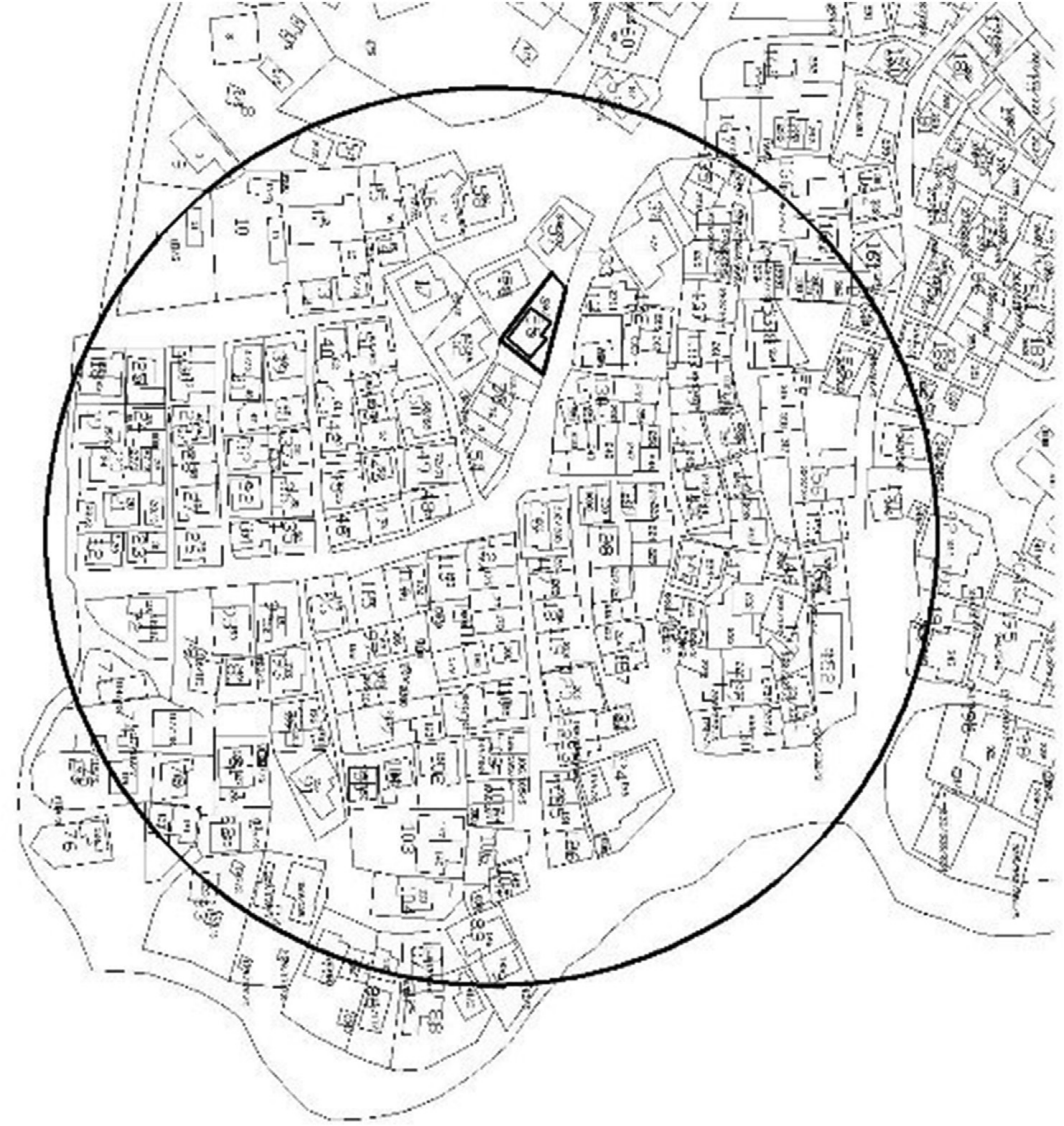
A spatial map of the study area (Setegn Meda). This map is accessed from Gulele Sub-City, Woredal 1, Administration Office, 2020. The map is the intellectual property of the sub-city and was used herein with permission.

**Figure 7:**
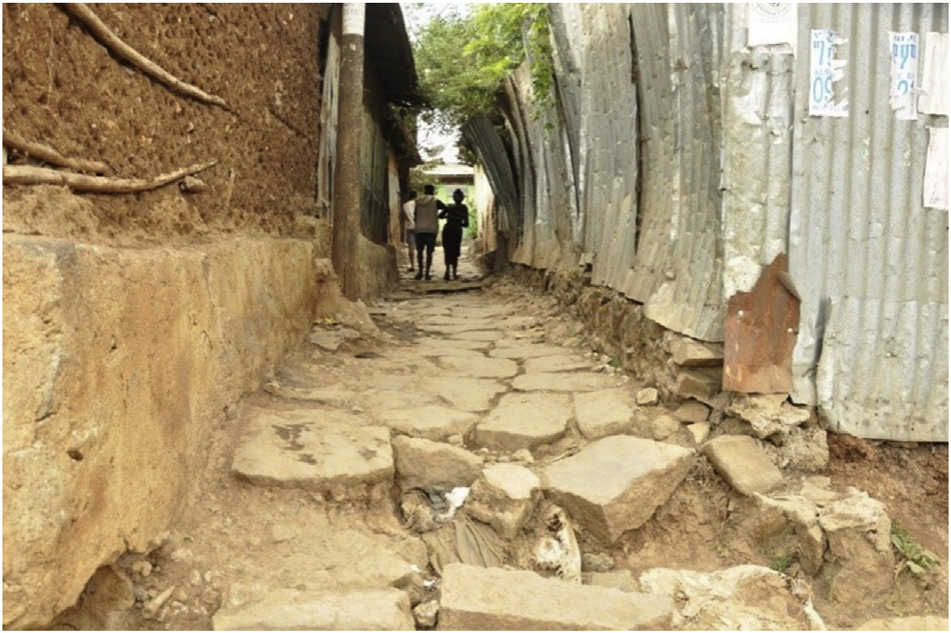
A typical narrow lane in the study area. This image was taken during field observation in February 2020.

The third morphological fact this study has identified is demography. The theme demography constitutes two sub-themes. The first sub-theme was *density*. From the field observation, one thing that can be said about the studied slum neighborhood is that it was a congested settlement. Despite the sub-standard materials they were made of, houses in the study area were built so close to each other. Pieces of parking lots, playgrounds and open spaces for social gatherings were rarely available. Most allies were very narrow. Figure 5 presents a panoramic view of a piece of congested settlement in the study area. Figure 6 presents a spatial map of the study area. Whereas, Figure 7 displays a typical narrow lane in the study area.

The second sub-theme was *homogeneity*. This study saw that the studied neighborhood was homogenous in two aspects. On one hand, it was homogenous in terms of religion. From the total number of participants, 18 were Christians. An informant (resident) also told, *“Most of the residents of this neighborhood are Christians.” (Meaza).* On the other hand*,* it was homogenous in terms of ethnicity. An informant (resident) mentioned, *“The majority of these residents are Amhara.” (Abiy).*

#### 4.2.4 Positionality

The positionality of the neighborhood is the fourth morphological fact. The theme positionality constitutes three sub-themes. The first sub-theme was *relative distance from the nucleuses of the city*. The field observation indicated that the neighborhood is located in a part of the city relatively distant from the nucleuses of the city. An informant (resident) reported, *“We need to travel a bit longer to access essential social services such as market, school, church, government organizations, etc.” (Musa).*

The second sub-theme was *relative distance from the center of the city (CBD)*. The field observation showed that the neighborhood is located in a part of the city relatively distant from the central business district of the city. The CBD of Addis Ababa is a mega business area known as Mercato. Residents have to travel at least 6 km to access Mercato.

The third sub-theme was *geographic location in the city*. The field observation indicated that the neighborhood is located in the northern part of the city. The field observation also indicated that the northern part of the city is bounded by a mountain range.

#### 4.2.5 Being a Slum as a Relational Construct

The last theme that emerged from the analysis of data associated with morphological facts is *Being a Slum as a Relational Construct*. This theme was made up of three sub-themes. The first sub-theme was *Being a Slum to the City Administration*. Interview data suggest that being a slum has two meanings to the City Administration. One, a place that comes last when it comes to the provision of public services. Two, a habitat that needs to be cleared and transformed after relocating the residents. For instance, an informant (resident) said, *“The city administration does not care whether we have access to public services or not.” (Megersa).* Another informant (resident) also told, “*The government only wishes to clear such types of neighborhoods and construct huge buildings for business.” (Mohamed*).

The second sub-theme was *Being a Slum to Non-Slum Dwellers*. Interview data suggest that, for non-slum dwellers, a slum is a place where economically, socially, and morally low standard people live. For instance, an informant (resident) mentioned, *“non-slum dwellers think people from slum areas are potential thieves.” (Abezash)*. Another informant (resident) also said, *“non-slum dwellers think people from slum areas often lack manners.” (Birtukan*).

The third sub-theme was *Being a Slum to the Inhabitants*. Interview data suggest that residents assume their neighborhood is unfavorable to settle and raise children. Interview data also suggest that residents assume their neighborhood as a transitionary settlement to life in a better place. For instance, an informant (resident) said, *“The neighborhood is not safe for children.” (Hagos).* Another informant (resident) also reported, *“I feel like I am living here until I move to somewhere better.” (Bulcha*).

### 4.3 Dysfunctions of morphological facts

Five major themes emerged from the analysis of interview and observation data about dysfunctions of morphological facts. These were: Physical Health Risk; Psychological Distress; Economic Cost; Social Cost; and The Gender Dimension of Dysfunctions. Table 3 presents the themes, sub-themes, and codes that emerged concerning dysfunctions of morphological facts.

**Table 3:** Themes, sub-themes, and codes related to dysfunctions of morphological facts.

Themes	Sub-themes	Codes
Physical Health Risk	Predisposition to Illness	Bacterial, viral, parasitic, allergy, traffic accident
	Predisposition to Health Risk Behavior	Drug abuse
Psychological Distress	Experiencing Negative Emotions	Feeling of Insecurity
		Anger, grievance and, sense of antagonism
		Frustration
	Negative Attitude towards one's Place of Residence	‘Leave this place whenever you get any opportunity’ mentality
Economic Cost	Incurring Extra Costs	Buying bottled water Traveling to nucleuses and center of the city
	Resource Migration	The outward flow of human or financial resource
	Obstruction of Livelihood Activity	Unable to carry out power-dependent livelihood activity
Social Cost	Differential Treatment by the City Administration	Differential distribution of solid waste facilities and human resources Taking a longer time to dispose of solid wastes Differential treatment of compliant reports
	Less Concerned City Administration	The city government is more interested in spaces that are hotspots of socio-economic and political activities The neighborhood is not a hotspot
	A Constraint to be a good Student	Unable to study at night due to irregular power blackout No space at home for a study room
	Crime	Hideaway for petty criminals
	Domestic Sexual Assault	Sharing bedroom No private space to change clothes
	Hardship to Administer an Emergency Response.	Narrow allies to drive through ambulance or firefighter vehicle.
The Gender Dimension of Dysfunctions	Women Suffer More	Women suffer more from the dysfunctions of Inadequate access to water and Non-proportional family size and number of bedrooms ratio.

#### 4.3.1 Physical health risk

One of the themes that emerged as the dysfunctions of morphological facts was a physical health risk. Two sub-themes had also emerged under this theme. The first sub-theme was *Predisposition to Illness*. The interview data suggest that four aspects of morphological facts predispose residents to various kinds of Illness: sub-standard construction materials of houses; unhygienic living conditions; the problem of municipal solid waste management; and the congested nature of the study area.

While describing the interior of their houses, informants (residents) mentioned that the mud-floor has been creating favorable conditions for insects (fleas, bedbug, and cockroach) to easily incubate and for rodents (mice and rats) to easily boreholes and nest. The government officials had also mentioned insects and rodents in relation to the inner features of the houses. For instance, one of the government officials (an expert from Gulele Sub-City, *Woreda* 1, Housing and Urban Development Office), said, *“…the mud-floor of the houses has doomed the people to live with rats and fleas”*. Science tells that living in such condition predispose people to various diseases that are transmitted by insects and rodents.

This study has also found out that the unhygienic living condition has been predisposing residents to different types of diseases. Informants reported that more than one member of their family has contracted a disease that doctors attributed to inadequate domestic hygiene. Some of these diseases mentioned by the informants were: bacterial (food poisoning, diarrhea, and skin infection); viral (gastroenteritis, colds, and flu); and parasitic (giardiasis, scabies infection, pediculosis, hookworm infection, threadworm infection, and Strongyloides). For instance, an informant (resident) said, *“I was once hard hit by diarrhea. Having prescribed the necessary medication, the doctor told me that I would not be sick if my living environment had been clean.” (Ketema).* Another informant (resident) also said, *“My children usually suffer from bacterial infections. Whenever I go to a clinic, they tell me that keep your children in a hygienic environment otherwise the illness will recur in no time.” (Senayit).*

This study has also found out that the problem of municipal solid waste management in the study area has been presumably predisposing residents to certain types of illnesses. For instance, some informants reported that there were family members who contracted respiratory tract allergies which doctors attributed to the pungent smell that emanates from solid waste dumpsites. Case in point, one informant (resident) reported, *“I am asthmatic. The pungent smell of solid wastes thrown on open spaces of the neighborhood is killing me.” (Lema).* Another informant (resident) also told*, “I usually know it when my sines start to resurrect the moment a bad smell of solid waste thrown on the roadsides creeps up my nostril. Sometimes the pungent smell of garbage piled up somewhere outside even comes to your home.” (Tsehay*).

Results suggested that the congested nature of the study area also predisposes children to traffic accidents. Informants reported that for there was no playground in the neighborhood children had been forced to play on roads and alleys exposing themselves to traffic accidents. One informant (resident) told, *“I have lost my 10 years old cousin in a traffic accident. My cousin was playing football on the roadsides when the accident happened.” (Dergu*).

The second sub-theme that emerged was *Predisposition to Health Risk Behavior*. The absence of playgrounds due to the congested nature of the settlement was also reported as one of the factors that predispose youths to health risk behavior such as substance abuse. One of the government officials (an expert from Gulele Sub-City, Woreda 1, Social Affairs Office Office) said, “*It is common that youths in the neighborhood spend their afternoons chewing khat. They would have spent their time playing football or any other sport if they had a playground.”*

#### 4.3.2 Psychological distress

The second theme that emerged as a dysfunction of morphological facts was psychological distress. This theme constitutes two sub-themes. The first sub-theme was *Experiencing Negative Emotions*. Interview data suggest that the perceived meaning of slums for the city administration (*A habitat that needs to be cleared and transformed after relocating the residents*) induces the feeling of insecurity. An informant (resident) said, *“I am always afraid that one day the government may displace us in the name of slum redevelopment.” (Samrawit)*.

Interview data also suggest that the meaning of slum for non-slum dwellers (*A place where economically, socially, and morally low standard people live*) induces feelings of anger, grievance and, a sense of antagonism. For instance, an informant (resident) told, *“The presumption people from non-slum dwellers have towards people from slum neighborhood is annoying. Sometimes their perception towards us makes me hate them.” (Terhas)*.

Interview data also suggest that sub-standard construction material and undependable access to basic infrastructures induce the feeling of frustration. Most interviewees (residents) reported that walls and roofs often leak during heavy rain despite their attempt to prevent it through plastic sheets. Relatedly, an informant (resident) mentioned, *“I always cry whenever the roof leaks after a repair. What can I do other than crying? If I had the money, I would have completely changed the old roof with a new one.”(Beza)*. Most interviewees (residents) also reported that the undependable access to tap water has made it difficult to keep domestic and personal hygiene. Relatedly, an informant (resident) said, *“…sometimes it is deeply sorrowing to not have water to wash your body. This feeling intensifies the moment you realize it is not that much easy to change your neighborhood.”(Nigist)*.

The second sub-theme that emerged under the theme labeled psychological distress was a *Negative Attitude towards one's Place of Residence.* Under section 4.2, there is a sub-section that discusses ‘*Being a Slum to the Inhabitants’.* In this section, it is mentioned that residents assume their neighborhood is unfavorable to settle and raise children. It is also mentioned that residents assume their neighborhood as a transitionary settlement to life in a better place. Analysis of interview data about dysfunctions of morphological facts further showed that ‘*Being a Slum to the Inhabitants’* results in a negative attitude towards one's place of residence characterized by ‘Leave this place whenever you get any opportunity’ mentality. For instance, an informant (resident) mentioned, *“I feel like I am living here until I move to somewhere better.” (Bulcha).* Such psychological tendency could probably demotivate residents from investing in their neighborhood.

#### 4.3.3 Economic cost

The third theme that emerged as a dysfunction of some morphological facts in the study area was economic cost. This theme has also emerged with three sub-themes. The first sub-theme was *Incurring Extra Costs.* Interview data suggest that residents in the study area incur a significant amount of money for two reasons. First, due to the undependability of tap water, residents were being forced to buy bottled water. Second, due to the relatively long distance, they need to travel to access essential public services located in the nucleuses and center of the city. For instance, an informant (resident) said, *“most of the time, we are being forced to buy bottled water due to the constant fluctuation of access to tap water.” (Ketema)*. Another informant (resident) also said, *“most essential services are located relatively far from this neighborhood, unlike others living in the centers of the city, we need to pay more to travel and access those services.” (Dergu)*.

The second sub-theme was *Resource Migration*. Interview data suggest that the meaning residents associate with their neighborhood (Being a Slum to the Inhabitants) has a tendency to push away potential human and financial resources residents accumulate over time. For instance, an informant (resident) mentioned, *“Whenever people make some money or get a better education, they start planning to leave this place.” (Senayit).* The resource migration appears to be one of the factors that hamper the possibility of future investment in the study area.

The third sub-theme was *Obstruction of Livelihood Activity*. Interview data suggest that the undependability of electric power has been challenging those whose livelihood activity heavily relies on electric power. For instance, an informant (resident) said, *“I feed my children by making and selling Enjera…I use an electric stove to make Enjera…The fluctuation of electric power has made my livelihood very challenging.” (Terhas).*

#### 4.3.4 Social cost

Social cost has also emerged as a dysfunction of some of the morphological facts in the study area. The theme-social cost constitutes six sub-themes. The first sub-theme was *Differential Treatment by the City Administration.* Results suggest that differential treatment by the city administration is manifested in three instances. First, it seems that the concerned government bodies address differentially power and tap water-related compliant reports from slum and non-slum neighborhoods. The interview data showed that concerned government bodies in the city were passive to provide a timely response to either power or water-related problems reported by residents from slum neighborhoods. Informants (residents) had told that electric power and water bureaus both at *woreda* and sub-city level were rather active in providing prompt response to problems reported from non-slum neighborhoods-especially those neighborhoods where rich people live. Second, as compared to non-slum neighborhoods in the city, the number of garbage containers of all sizes and janitors in the study area were found out to be at the lowest levels. Only two medium-size waste containers were observed. As for the number of street cleaners, participants have reported that only two women were often observed doing the cleaning job. One informant, *(Mohamed)*, described this situation saying, *“Walk around Bole area, you will find residential garbage containers at every corner but you will find none in our entire neighborhood…Walk around in Bole area at* 5 AM *in the morning, you will find many janitors cleaning main roads, streets, and alleys but I hardly believe that you will find one in our entire neighborhood*.”. An expert from Gulele Sub-City, Woreda 1, Administration Office also said*, “From my observation, I have seen that the distribution of waste containers and street cleaners in the city is disproportional. Non-slum neighborhoods account for the highest number of both the facility and the human resource”.* Three, the field observation has made clear that solid wastes generated at the household level were temporarily stored in an open space. Besides, informants (residents) reported that it usually takes 15–30 days for the municipality to collect solid wastes from the open spaces and properly dispose of. An informant (resident) mentioned, *“The government would not take much longer time to collect solid wastes from the neighborhood and properly dispose of it if the neighborhood were not a slum neighborhood.” (Abezash)*.

The second sub-theme was *Less Concerned City Administration*. In our previous discussion about a morphological fact-*positionality*, it is mentioned that the neighborhood is located in the northern part of the city. It is also mentioned that the northern part of the city is bounded by a mountain range. It seems due to the mountain bounded nature of the northern part of the city, the city was rather horizontally expanding in directions never bounded by mountain ranges (the eastern, western, and southern directions). The rapid expansion of the city in the eastern, western, and southern directions and the related socio-economic and political problems seem to have caught the attention of the city administration. This is probably the city government is more interested in spaces that are hotspots of socio-economic and political activities. Unfortunately, due to its geographic location in the city, the neighborhood seems not to be a hotspot for socio-economic and political activities. This in turn seems to have made the city administration to be less concerned about the neighborhood. For instance, an expert from *Woreda 1*, Housing and Urban Development Office said, *“Two parts of the city often concern the city administration. One is the inner-city slum. And the other is the fringe of the city that is rapidly sprawling and inducing political tension between the Addis Ababa city Administration and the regional state government of Oromia.”* The fact that the city administration is less concerned about the study area could probably be a factors that hamper the possibility of future investment in the neighborhood.

The third sub-theme was *Constraint to be a good Student*. Interview data suggest that students in the neighborhood suffer from dysfunctions of two morphological facts. First, due to undependable access to electric power, students sometimes are unable to study or do home-works at night. Two, due to the non-proportional family size and bedroom ratio, most students do not get a quiet and separate space to study at home. For instance, an informant (resident) said, *“I have three children living in a single bedroom house. Two of them are high school students. They study in the living room where everybody moves her and there. I often notice how hard it has been for them to study at home.” (Terhas)*.

The fourth sub-theme was *Crime.* One of the dysfunctions of the congested nature of the studied neighborhood was that it serves as a perfect hideaway for petty criminals. Informants (residents) reported that after petty criminals (shoplifters, muggers, snatchers, pickpockets, etc.) have stolen something, often run into the neighborhoods to escape.

The fifth sub-theme was *Domestic Sexual Assault*. Based on the information gathered from the government officials, it could be presumed that one of the dysfunctions of non-proportional family size and number of bedroom ratio could be the predisposition of teenage girls to domestic sexual assault. For instance, one of the government officials (an expert from Gulele Sub-City, Woreda 1, Social Affairs Office Office), mentioned that his office has records of three sexual assaults against teenage girls from the study site perpetrated by their closest relatives. This official had also mentioned that all of those girls were either used to share a bedroom with their perpetrator or had no secured private space to change their clothes and/or sleep.

The sixth sub-theme was *Hardship to Administer an Emergency Response*. One of the dysfunctions of the narrowness of roads and lanes in the study area was that it has made the delivery of emergency responses so hard. The field observation showed that the allies are too narrow to drive through an ambulance or firefighter vehicle. Relatedly, one of the government officials (an expert from Gulele Sub-City, Woreda 1, Administration Office) said, *“It would not be easy to navigate through the neighborhood to provide emergency responses for potential urban fire… the houses are built on one another, pathways and alleys are very narrow…if there is fire, the whole thing will burn out.”* An informant (resident) also said, *“For the lanes are too narrow, I have seen people carrying a pregnant woman towards an ambulance parked somewhere else.” (Birtukan).*

#### 4.3.5 The gender dimension of the dysfunctions

This study found that the dysfunctions of morphological facts have gender dimensions. According to this study, women suffer more from some dysfunctions of morphological facts. For instance, women were observed suffering more from the dysfunction of inadequate access to tap water. Informants (residents) reported that the irregularity of access to water has been compromising the efforts made to keep personal hygiene. In this regard, this study has found that the irregularity of water affects women more than men. Case in point, one informant *(female, age 25)*, reported, *“The irregularity of water is not a problem for men. Because they can easily go to those places where one can take a shower for 10–15 Ethiopian Birr. But I can't do that. It is uncommon for a woman to shower in such places.”*

Females (teenage girls) were also observed suffering more from the dysfunction of non-proportional family size and number of bedroom ratio. In a previous discussion, it is mentioned that, unlike boys, the non-proportional family size and number of bedroom ratio potentially predisposes teenage girls to domestic sexual assault. Relatedly, an informant (resident) said, *“I live with my three teenage children in a single bedroom house. Living in such condition is uncomfortable for my girls. It might be even risky for them.” (Samrawit)*.

## 5 Discussion

Five major themes emerged from the analysis of interview and observation data about morphological facts. These were: Dilapidated Housing; Limited Access to Infrastructure; Demography; Positionality; and Being a Slum as a relational Construct. Five more major themes also emerged from the analysis of interview and observation data about dysfunctions of morphological facts. These were: Physical Health Risk; Psychological Distress; Economic Cost; Social Cost; and The Gender Dimension of Dysfunctions.

To locate this study among the extant literature on slum settlement, the findings were compared against findings of other related researches. The comparison shows that much of the findings of this study appear in agreement with the findings of several previous studies. For instance, consistent with the findings of this study, previous studies have shown that housing condition in slum neighborhoods of Addis Ababa city has been very poor. UN-Habitat, (2007a, b), documented that most houses in slum neighborhoods of Addis Ababa were made of sub-standard construction materials where most residents suffered from leakage during rainy seasons. Some studies have also shown that the general condition of slum neighborhoods has been unhygienic (Biniyam et al., 2018; Getachew et al., 2007; Sanbata et al., 2014; Tolossa, 2010; and UN-Habitat, 2007a, b). Tolossa (2010), on the other hand, has described how the housing condition in the slum neighborhood of Addis Ababa was poor by implicating the non-proportionality of family size and bedrooms ratio.

Regarding access to basic infrastructures, plenty of studies showed that residents of slum neighborhoods in Addis Ababa city have inadequate access to basic infrastructures such as electricity, tap water, and solid waste facilities (Biniyam et al., 2018; Tolossa, 2010; UN-Habitat, 2007a). Studies have also documented different problems of lack of sufficient access to basic infrastructures. For instance, Biniyam et al. (2018); Getachew et al., and (2007); and Tolossa (2010), wrote that lack of sufficient access to basic infrastructures (such as sanitation facilities and water) in slum neighborhoods of Addis Ababa city has been predisposing residents to different types of communicable diseases.

Findings concerning the congested nature of settlement patterns and their dysfunctions also appeared in convergence with previous studies. Tolossa (2010); and UN-Habitat, (2007a, b), showed that slum neighborhoods in Addis Abba city were densely populated. The problems of congested urban settlements such as deviance and crime are also well documented in urban sociology texts (Gottdiener, 2019).

Studies on the effect of positionality have documented that a neighborhood's relative distance from centers of the city has different economic ramifications (Brueckner and Rosenthal, 2009; Heikkila et al., 1989; Van Ham et al., 2013; Weber and Kwan, 2003). Related studies have also documented the impact of geographic location in the city on residents of a given urban neighborhood (Dietz, 2002; Friedrichs et al., 2003; Van Ham et al., 2013; Weber and Kwan, 2003).

This study found that being a slum to the city administration is being a place that needs to be cleared. Whereas, being a slum to non-slum dwellers is being a place where economically, socially, and morally low standard people live. Consistent with this study, previous studies have also claimed that city administrations tend to see slums as badlands where the only solution is slum clearance (Arimah, 2001; Gilbert, 2007; Milbert, 2006). Other studies have also mentioned that people from non-slum areas have a negative attitude towards people living in slums (Goswami and Manna, 2013; Roitman, 2005; Skogan, 1986).

This study also found that the dysfunctions of morphological facts have a gender dimension. According to this study, women suffer more from the dysfunctions of some morphological facts. Agreeable to this study, related studies have also reported that the distribution of infrastructure differentially affects men and women (Crow and Sultana, 2002; Kher et al., 2015; Parikh et al., 2015).

At this point, it is worth reminding Durkheim's theory of social facts. Durkheim argues that social facts are *‘sui generis’* realities above and beyond the individual and should not be reduced to economic, psychological, or physiological facts (Durkheim, 1966). Guided by this theoretical understanding, throughout this paper, dysfunctions are conceptualized as *‘sui generis’* realities (social facts) and explained in terms of other ‘facts’ (morphological facts) than through psychological, economic, or physical characteristics of the individuals residing in a slum neighborhood.

It is also worth reminding the two basic arguments mentioned in the introductory part and locate this study right where it belongs. These two arguments are one an argument involving the use of the term ‘Slum’ and the other is concerning the homogeneity/heterogeneity of slums. Behind the argument on the use of the term ‘slum’, there are two camps. One constitutes the United Nations, World Bank, and other international development organizations, and orthodox academicians who took the term for granted and utilizes it in its simplistic form. Whereas the other camp constitutes critical intellectuals who saw the danger in using the term as it has been used today (Gilbert, 2007). The critical camp argues that the word ‘slum’ is dangerous for it creates favorable conditions for unscrupulous politicians, developers, and planners to manipulate the term for their benefits against the expense of the slum dwellers (Gilbert, 2007). It also has a misleading effect on policymakers to uphold false hopes that a campaign against slums raise and to cling to a dangerous solution like ‘slum clearance’ (Gilbert, 2007).

This study senses some empirical evidence that adds a point to the critical camp. Case in point, the government informants interviewed for this study seem to share a mentality that the slum in the city is a ‘badland’ that needs to be cleared. This mentality is nothing but a manifestation of how slums are conceptualized in and through policy frameworks. Above and beyond, Weldeghebrael (2020), has well stated how slum redevelopment is framed by the developmental state government of Ethiopia to facilitate the capital accumulation and consolidate its tight grip on power.

As in the case with the use of the term ‘slum’, observers also debate on the heterogeneity/homogeneity of slum habitat. On one hand, giant international organizations such as the United Nations and World Bank, politicians, planners, and the general mass tend to portray all slums as bad and everyone living in them suffering from their grim realities (Gilbert, 2007). On the other hand, plenty of observers have also put forward their counter-arguments (Gilbert, 2007; Stokes, 1962; Turner, 1967).

If the findings of this study seen from the heterogeneity/homogeneity argument, it could be understood that the morphological facts in the study area may tell stories of the difference and similarity between the study area and other slum neighborhoods. In this regard, a comparison based on some morphological facts suggests that the study area is somehow different from other forms of slums mentioned in Alemayehu (2008). For instance, unlike old inner-city slums, the study area is relatively an emerging settlement whose current residents started settling around 1991. Unlike the inner-city squatter plastic housings, the housing units in the study area are made from wood, mud, and corrugated iron. Again, unlike the informal peripheral settlements, the settlement in the study area is a formal settlement situated neither in the inner-city nor at the periphery of the city. Table 4 presents a comparison between the study area and forms of local slum areas outlined by Alemayehu (2008).

**Table 4:** A comparison between the study area and other forms of local slum areas outlined by Alemayehu (2008).

Morphological Fact	Description	Types of Slums			
		Types of Slums in Addis Ababa as outlined by Alemayehu (2008)			
		The study area	Non-planned old inner-city settlements	Informal peripheral squatter settlements	Inner-city squatter plastic houses
Construction Material (*Mud wall and Corrugated iron roof*)	Yes	✓	✓	✓	
	No				✓
Settlement Pattern (*In terms of planning*)	Formal				
	Informal	✓	✓	✓	✓
Settlement Status *(In terms of legality)*	Legal	✓	✓		
	Illegal			✓	✓
Settlement Status (*In terms of land tenure security)*	Secure				
	Slightly Secure	✓	✓		
	Insecure			✓	✓
Settlement Pattern (*In terms of temporal dimension)*	Permanent	✓	✓	✓	
	Temporary				✓
Distance (*Relative distance from nucleuses of the city)*	Proximal		✓		✓
	Adjacent	✓			
	Distant			✓	
Distance (*Relative distance from the center of the city or CBD)*	Proximal		✓		✓
	Adjacent				
	Distant	✓		✓	
Geography (*Located in the part of the city that is bounded by a Mountain)*	Yes	✓			
	No		✓	✓	✓
Geographic Location in the City	Inner-city		✓		✓
	Transition Zone	✓			
	Periphery			✓	
Age	New (<10 years)			✓	✓
	Emerging (10–30 years)	✓		✓	✓
	Old (>30 years)		✓		

Table 4 suggests that let alone the homogeneity of slums at the global level, slums are rather heterogamous even at a national/local level. Further exploration of the typologies of slums in Addis Ababa as proposed by Alemayehu (2008) suggests reconsideration of the classification of slums due to the following reasons. One, his classification confuses informality with illegality. Two, his classification acknowledges slums neither in the inner-city nor on the periphery. Three, his classification does not account for variables that could make a qualitative and quantitative difference in life among slum settlements. Some of these variables could be a geographic location in the city; geography (being located in the part of the city that is bounded by a Mountain); distance (Relative distance from nucleuses or CBD of the city), etc.

## 6 Conclusion

Drawing data from interviews and field observation and borrowing theoretical constructs from the functionalists’ perspective, this study, tried to address three specific research objectives. (1) To explore morphological facts that typically characterize life in *Setegn Meda* slum neighborhood. (2) To identify dysfunctions of those morphological facts. (3) To discuss some of the differences and similarities between the study area and other forms of a slum in Addis Ababa as outlined by Alemayehu (2008) based on selected morphological facts.

The study found that the morphological facts that typically characterize life in *Setegn Meda* slum neighborhood are: Dilapidated Housing; Limited Access to Infrastructure; Demography; Positionality; and Being a Slum as a relational Construct. The study also found that these morphological facts have their inherent dysfunctions: Physical Health Risk; Psychological Distress; Economic Cost; and Social Cost. Results further show that the dysfunctions of some morphological facts have a gender dimension where women suffer more.

The difference and similarities between the study area and other forms of a slum in Addis Ababa as outlined by Alemayehu (2008) were discussed based on selected morphological facts. The discussion implies that let alone the homogeneity of slums at the global level, slums are rather heterogamous even at a national/local level. The discussion has also highlighted that the classification of slums in Addis Ababa as proposed by Alemayehu (2008) for one thing is not exhaustive and needs some modifications.

The study is expected to hold significance both at the empirical and theoretical levels. The empirical significance of this study, inter alia, are: it has challenged the conventional categorization of slums in Addis Ababa; it has provided a thick description of morphological facts in a slum neighborhood and their dysfunctions to the inhabitants, and it has brought the gender dimension of the dysfunctions of morphological facts to the audience.

The theoretical significance of this study could be drawn from how it tried to make functionalism theoretically useful to the study of slum habitats. As mentioned in section two of this paper, this study rooted its theoretical foundation into two basic concepts drawn from the functionalist literature- *‘Morphological Fact’* and *‘Dysfunction’*. By borrowing the concept of morphological fact, the study: One, tried to capture most of the sociological dimensions of ‘space’. Two, has recognized and framed the physical conditions of life in a slum neighborhood as social facts of a morphological order (morphological facts). Three, has understood and elaborated the morphological facts of life in a slum neighborhood and their dysfunctions as *sui generis* realities that are irreducible to ecological, economic, and psychological factors.

Whereas, by borrowing the term dysfunction in Merton's sense and integrating it with Durkheim's conception of morphological fact, this study: One, has framed the negative consequences (dysfunctions) of physical aspects of life in a slum neighborhood (morphological facts) within a sociological paradigm. Two, has conceptualized dysfunctions of morphological facts in a slum neighborhood as emergent properties irreducible to psychological, economic, or physical characteristics of the individuals residing in a slum neighborhood.

## Declarations

### Author contribution statement

Edomgenet Hiba Issa: Conceived and designed the experiments; Performed the experiments; Analyzed and interpreted the data; Contributed reagents, materials, analysis tools or data; Wrote the paper.

### Funding statement

This research did not receive any specific grant from funding agencies in the public, commercial, or not-for-profit sectors.

### Data availability statement

Data will be made available on request.

### Declaration of interests statement

The authors declare no conflict of interest.

### Additional information

No additional information is available for this paper.

## Footnotes

1 *Woreda* refers to the lowest level of an administrative entity in the bureaucratic hierarchy of Addis Ababa city administration.

2 *Kebele* houses are low-cost rental houses that are built by the government for the very poor households.
